# Novel Spatially Asymmetric Copper Bismuthate‐Mediated Augmentation of Energy Conversion to Realize “Three‐Step” Tumor Suppression

**DOI:** 10.1002/advs.202402599

**Published:** 2024-04-23

**Authors:** Jiarui Wang, Haoqin Zheng, Guangyao Hu, Xujian Yang, Hongpeng You, Lile Dong, Shuyan Song

**Affiliations:** ^1^ Key Laboratory of Rare Earths Chinese Academy of Sciences Ganjiang Innovation Academy, Chinese Academy of Sciences Ganzhou 341000 China; ^2^ School of Rare Earths University of Science and Technology of China Hefei 230026 China; ^3^ State Key Laboratory of Rare Earth Resource Utilization Changchun Institute of Applied Chemistry Chinese Academy of Sciences Changchun 130022 China

**Keywords:** CuBi_2_O_4_, sonodynamic therapy, sonosensitizers, spatially asymmetric, tumor suppression

## Abstract

The generally undesirable bandgap and electron–hole complexation of inorganic sonosensitizers limit the efficiency of reactive oxygen species (ROS) generation, affecting the effectiveness of sonodynamic therapy (SDT). Comparatively, the novel polyvinylpyrrolidone‐modified copper bismuthate (PCBO) sonosensitizers are manufactured for a “three‐step” SDT promotion. In brief, first, the strong hybridization between Bi 6s and O 2p orbitals in PCBO narrows the bandgap (1.83 eV), facilitating the rapid transfer of charge carriers. Additionally, nonequivalent [CuO_4_]^6−^ layers reduce crystal symmetry, confer PCBO unique piezoelectricity, and improve electron–hole separation under ultrasonic (US) excitation. This allows PCBO to convert US energy into chemical energy to produce ROS, achieving the accumulation of abundant ROS, resulting in apoptosis and tumor suppression. Concurrently, PCBO also acts as a glutathione scavenger to reduce tumor antioxidant capacity and improve efficacy. To the best of authors understanding, this study reveals PCBO as an innovative piezoelectric sonosensitizer and provides a meaningful paradigm for designing energy conversion strategies for tumor suppression.

## Graphical abstract

The polyvinylpyrrolidone‐CuBi_2_O_4_ (PCBO) sonosensitizers are manufactured for enhancing sonodynamic therapy (SDT). PCBO possesses a small bandgap, facilitating the rapid transfer of charge carriers. And [CuO_4_]^6−^ are constructed to form spatially asymmetric structure, endowing PCBO with piezoelectric properties. PCBO can generate ·O_2_^−^ under ultrasound (US), resulting in mitochondrial damage to induce apoptosis, inhibiting the growth of tumor.

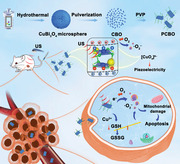

## 1 Introduction

Triple‐negative breast cancer (TNBC) features high aggressiveness, easy local recurrence, and distant metastasis. Although surgery, radiotherapy, and chemotherapy are the mainstream treatments for TNBC, susceptibility to recurrence and drug resistance result in low overall survival rates.^[^ ^1^, ^2^, ^3^ ^]^ Fortunately, the focus of multidisciplinary deep crossover is to design and construct novel biomaterial‐based cancer treatment strategies in preclinical settings, alleviating or even solving the shortcomings of traditional treatment strategies.^[^ ^4^, ^5^, ^6^, ^7^ ^]^ Sonodynamic therapy (SDT) assisted by sonosensitizers stands out for its noninvasiveness, desired biological tolerability, and high tissue penetration depth.^[^ ^8^, ^9^, ^10^ ^]^ Notably, several concurrent clinical trials (NCT05362409; NCT05580328) are evaluating the antitumor efficacy of sonosensitizer‐mediated SDT, either through monotherapy or synergistic treatment, signifying the substantial clinical promise of SDT. Indeed, ultrasound (US) facilitated sonosensitizers to interact with the surrounding molecules to generate abundant reactive oxygen species (ROS), disrupting the intracellular balance of oxidative stress and inducing apoptosis of tumor cells.^[^ ^11^, ^12^ ^]^ However, the efficiency of ROS generation by excitation of acoustic sensitizers is often affected by insufficient conduction band potential, limited mobility of carriers, and rapid compounding. As a representative sonosensitizer, titanium dioxide undergoes the rapid recombination of electron–hole pair, leading to inefficient reactive oxygen species (ROS) production, along with the resistance of the antioxidative tumor microenvironment, the development of this strategy was restricted.^[^ ^13^, ^14^, ^15^, ^16^ ^]^

Intriguingly, a new class of sonosensitizers has received extensive attention to overcome the electron–hole pair recombination. Under US irradiation, the positive and negative ion centers in the piezoelectric sonosensitizers no longer coincide due to the spatially asymmetric structure, the charge balance is broken, and the internal electric field is formed by polarization.^[^ ^17^, ^18^ ^]^ Thus, piezoelectric sonosensitizers could easily generate a built‐in electric field and rapid inhibition of electron–hole pair recombination to drive redox reactions, further forming ROS.

Previous exploration on piezoelectric sonosensitizers dominantly concentrated on Bi_2_MoO_6_ (mm2),^[^ ^19^ ^]^ BaTiO_3_ (4 mm),^[^ ^20^ ^]^ and ZnO (6 mm).^[^ ^21^ ^]^ Interestingly, 20 of 32 crystallographic point groups possess potential as piezoelectric sonosensitizers, such as spatially asymmetric point group 4. Despite the unclear mechanism of SDT at the current stage and the SDT based on piezoelectric effect is still in its infancy, the design and construction of novel piezoelectric sonosensitizers provide a reliable direction for the exploration of the mechanism of SDT.^[^ ^22^, ^23^, ^24^ ^]^

**Scheme 1:**
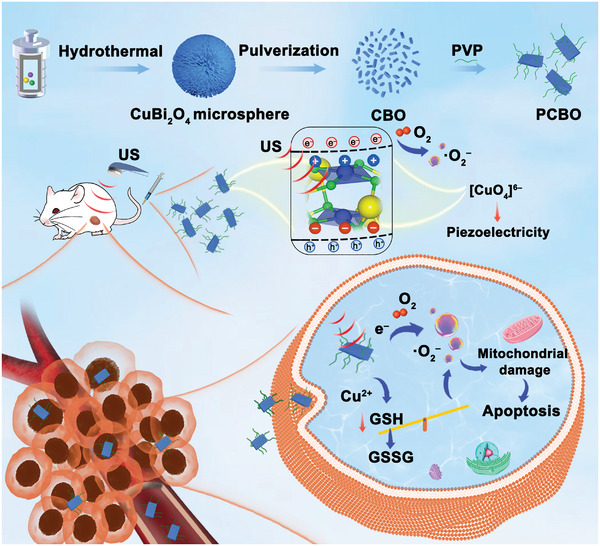
Schematic diagram of PCBO promotes energy conversion to realize “three‐step” tumor suppression.

Herein, we innovatively designed and constructed polyvinylpyrrolidone‐modified copper bismuthate (PCBO) piezoelectric sonosensitizers with small bandgap and spatially asymmetric point group 4 for enhancing tumor oxidative stress (**Scheme** **1**).Compared with the bandgap of TiO_2_ (3.20 eV), PCBO has the desired bandgap (1.83 eV), enhancing the migration of carriers. The strong hybridization between the Bi 6s and O 2p orbitals in PCBO piezoelectric sonosensitizers could reduce the bandgap, facilitating electron–hole separation. And nonequivalent Cu atoms in the unit cell exhibit a spatially asymmetric point group 4 by rotating along the *c*‐axis, reducing the crystal symmetry. Spatially asymmetric point group 4 endows PCBO with piezoelectric sonosensitizers performance, making their positive and negative charges deviate from the center in response to ultrasound (US) stimulation, inhibiting electron‐hole pair recombination. Inspiringly, PCBO possesses excited electrons and rapid charge transfer ability under US irradiation, ensuring the formation of abundant superoxide anion radical (·O_2_^−^), resulting in apoptosis and inhibiting the proliferation and growth of tumors. Excess ROS causes mitochondrial damage, activating apoptotic pathways. Importantly, PCBO possesses the ability to consume glutathione (GSH) based on the redox potential of Cu^2+^ ions, alleviating the antioxidant properties. Therefore, the novel PCBO piezoelectric sonosensitizers provide meaningful implications and guidance for the development of a simple and efficient energy conversion anticancer strategy.

## 2 Results and Discussion

### 2.1 Spatially Asymmetric PCBO

**Figure 1:**
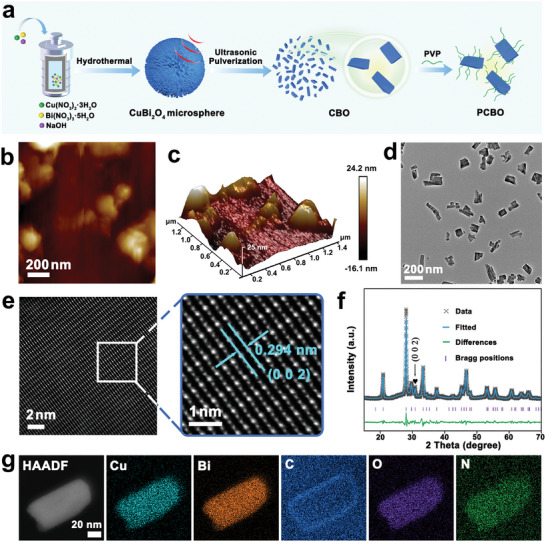
Synthesis and characterization of novel PCBO. a) Schematic diagram of synthesis PCBO. b) The atomic force microscopy (AFM) image and c) corresponding 3D image of PCBO. d) AC‐TEM image of PCBO. e) AC‐HAADF‐STEM image of PCBO. f) The XRD Rietveld refinement of PCBO. g) EDX element mapping images of PCBO.

The fabrication process of PCBO is presented in **Figure** **1**a. Briefly, CuBi_2_O_4_ microspheres with nanorods aggregated were prepared by hydrothermal method utilizing the Cu(NO_3_)_2_·3H_2_O, Bi(NO_3_)_3_·5H_2_O and NaOH as the precursors (Figures S1 and S2, Supporting Information). Subsequently, CuBi_2_O_4_ nanofragments (CBO) could be manufactured by ultrasonic pulverizing CuBi_2_O_4_ microspheres. Finally, PCBO was obtained with polyvinyl pyrrolidone (PVP) modification. Atomic force microscopy (AFM) (Figure 1b,c) and aberration‐corrected transmission electron microscopy (AC‐TEM, Figure 1d) images indicate the irregular morphology of PCBO. Aberration‐corrected high‐angle annular dark‐field scanning transmission electron microscopy (AC‐HAADF‐STEM) images displayed the arrangement of Bi and Cu atomic (Figure 1e). The lattice spacing was measured to be 0.294 nm, agreed with the (0 0 2） crystal plane of CuBi_2_O_4_ (PDF#97–001–2104). Interestingly, PCBOmaintains steady space group I4 belongs to the asymmetric point group 4, consistent with CBO and CuBi_2_O_4_ microspheres by X‐ray powder diffraction (XRD) patterns (Figure S3, Supporting Information). Additionally, the XRD Rietveld refinement of PCBO was carried out to reveal the pure crystalline phase with space group I4 (Figure 1f). The refined parameters fitted nicely to the calculations, confirming that the asymmetric point group 4 of CuBi_2_O_4_ (Table S1, Supporting Information). To visually observe the asymmetric point group 4, the representative (0 0 2) crystal plane is mentioned based on the XRD Rietveld refinement. As displayed in Figure S4 (Supporting Information), CuBi_2_O_4_ consists of [CuO_4_]^6−^ layers connected by Bi atoms, space group I4, in which the Cu atom is bonded to four equivalent O atoms and Bi is bonded to six O atoms. [CuO_4_]^6−^ layers constructed by nonequivalent Cu atoms rotationally stack along the *c*‐axis, forming a spatially asymmetric structure, which accounts for the piezoelectric properties of PCBO. The molar ratio of Cu and Bi was measured to be 1:2 by the inductively coupled plasma optical emission spectrometer (ICP‐OES), corresponding to the stoichiometric ratio. Furthermore, energy‐dispersive X‐ray spectroscopy (EDX) mapping images display that Cu, Bi, O, C, and N elements are distributed in the PCBO (Figure 1g).

**Figure 2:**
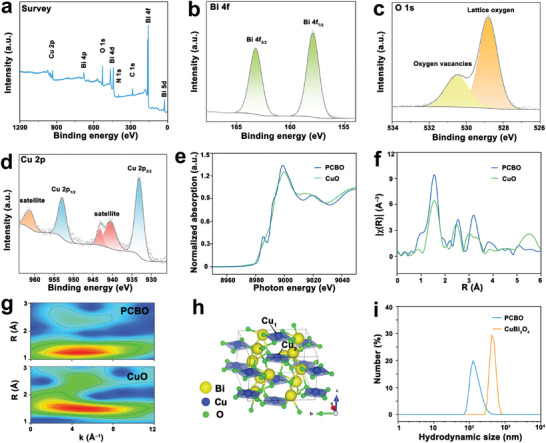
Chemical and coordination environment of PCBO. a) XPS spectra survey of PCBO. High‐resolution spectra of b) Bi 4f, c) O 1s, and d) Cu 2p. e) Cu K‐edge XANES spectra of PCBO and CuO referential sample. f) *k^3^*‐weighted *χ(k)*‐function Cu K‐edge EXAFS of PCBO and CuO referential sample. g) Wavelet transforms for the *k^3^*‐weighted Cu K‐edge EXAFS signals of PCBO and CuO referential sample. h) Spatially asymmetric structure sketch of PCBO. i) Hydrodynamic size of PCBO and CuBi_2_O_4_ microspheres.

Subsequently, the coordination environment of PCBO was systematically characterized. First, the composition and valence states were analyzed by X‐ray photoelectron spectroscopy (XPS). Briefly, the XPS survey spectrum confirming the presence of Cu, Bi, O, C, and N elements in PCBO (**Figure** **2**a), which is consistent with the results of the EDX mapping images. The two peaks of Bi 4f are located at 163.2 eV (Bi 4f_5/2_) and 157.9 eV (Bi 4f_7/2_), respectively, indicating the existence of Bi^3+^ ions (Figure 2b). The characteristic peaks of O 1s could be attributed to lattice oxygen and oxygen vacancies (Figure 2c).^[^ ^25^ ^]^ As exhibited in Figure 2d, the representative peaks of Cu 2p are Cu 2p_1/2_ (953.0 eV) and Cu 2p_3/2_ (933.1 eV), respectively, and the satellite peaks are attributed to the d^9^ configuration, revealing the existence of Cu^2+^ ions. Because nonequivalent Cu atoms occupy square coplanar [CuO_4_]^6−^ make PCBO have asymmetric point group 4, the fine coordination information of Cu element in PCBO was further evaluated by the X‐ray absorption near‐edge structure (XANES) and extended X‐ray absorption fine structure (EXAFS). The oxidation state of the Cu element was analyzed by the Cu K‐edge XANES curve, with CuO as a referential sample (Figure 2e). The similar position of the PCBO and CuO referential sample indicates the +2 oxidation state of the Cu element, which is consistent with the Cu 2p XPS analysis results. Subsequently, the local coordination of Cu sites was investigated from EXAFS analysis (Figure 2f). Both the EXAFS spectra of PCBO and CuO referential sample in R space display a similar characteristic peak, which is comparable to that of the Cu–O in CuO. The results of wavelet transform (WT) show the k^2^‐space most intense signal centered at 4.8 Å^−1^, consistent with that of CuO (Figure 2g), implying PCBO possesses a Cu–O coordination. Therefore, the PCBO features the asymmetric point group 4 based on the result of the AC‐HAADF‐STEM image, XRD Rietveld refinement, and EXAFS (Figure 2h). The modification of PVP was verified by the Fourier transform infrared (FTIR) spectrum (Figure S5, Supporting Information). For instance, the characteristic peak at 1660 cm^−1^ represents the vibration of the C═O bond, confirming the successful coating of PVP.^[^ ^26^, ^27^ ^]^ Furthermore, the amount of PVP on the surface of PCBO was ≈2.7% of the particle mass (Figure S6, Supporting Information). Compared with CBO, PCBO has long‐term aqueous dispersed stability (Figure S7a, Supporting Information). PVP can significantly improve the dispersion of PCBO in aqueous solutions (hydrodynamic size: 146 nm, Figure 2i; Figure S7, Supporting Information). In addition, the adoption of PVP can improve the retention time of PCBO in blood circulation, thus contributing to the passive accumulation in tumor tissues.^[^ ^28^, ^29^, ^30^, ^31^ ^]^

### 2.2 “Three‐Step” Promotion of PCBO

**Figure 3:**
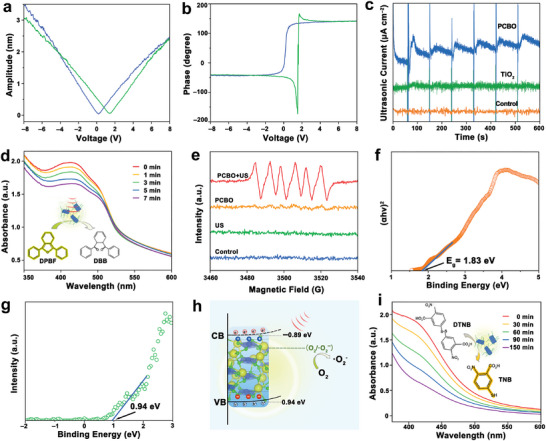
Piezoelectric SDT performance of asymmetric PCBO. a) The butterfly curve and b) phase spectrum of PCBO by the PFM characterization. c) Ultrasonic current density of PCBO, TiO_2_ and blank control group under US irradiation. d) Time‐dependent degradation of DPBF indicating ·O_2_^−^ generation. e) ESR spectra trapped by DMPO from PCBO + US, PCBO, US and control group. f) The inset shows the gap calculation of PCBO. g) VB spectra of PCBO detected by XPS. h) The mechanism of PCBO for piezoelectric SDT. i) UV–vis absorption spectra of DTNB, indicating time‐dependent GSH consumption by PCBO.

Piezoresponse force microscopy (PFM) test was conducted to detect the piezoelectric properties of asymmetric PCBO. As displayed in **Figure** **3**a, the butterfly curve of the hysteresis amplitude voltage could be observed, indicating PCBO has good piezoelectric performance. The phase spectrum of PCBO exhibits a phase difference obviously (Figure 3b), and the average phase contrast is ≈180°, revealing the piezoelectric polarization switching process. Then, the current generation performance of PCBO was evaluated systematically under US (Figure 3c). The ultrasonic current signal can be clearly observed and PCBO exhibited a stronger ultrasonic current signal than that of titanium dioxide and blank control group,^[^ ^32^ ^]^ indicating that more US‐excited electrons were generated at the surface of PCBO. Electrochemical impedance spectroscopy (EIS) of PCBO and TiO_2_ was also determined, and PCBO exhibited a smaller arc compared to titanium dioxide, suggesting lower impedance and faster charge migration (Figure S8, Supporting Information), coinciding with the recovery of ultrasonic currents.

Inspiringly, the SDT performance of PCBO was validated by a series of in vitro solution tests. First, the ROS types of PCBO were investigated using the 1,3‐diphenylisobenzofuran (DPBF) and 5,5‐dimethyl‐1‐pyrroline‐N‐oxide (DMPO) probe. For instance, as displayed in Figure 3d, the absorbance in the PCBO group decreases with increasing duration of US compared with the control group (Figure S9, Supporting Information), which is attributed to the epoxidation of ·O_2_^−^ with DPBF and obtaining colorless 1,2‐dibenzoylbenzene (DBB). Interestingly, as exhibited in Figure 3e, there are distinctive peaks ·O_2_^−^ in the PCBO+US group, while those of the control, US, or PCBO group are inconspicuous. The bandgap energy (Figure 3f) and the valence band (VB) position (Figure 3g) of PCBO were determined to be 1.83 and 0.94 eV, respectively, and the conduction band (CB) was −0.89 eV. Interestingly, the bandgap energy of PCBO was found to be narrower compared to other representative piezoelectric sonosensitizers (Table S2, Supporting Information), which can facilitate ROS generation by US irradiation. Comprehensively, the potential ·O_2_^−^ generation mechanism of SDT based on the spatially asymmetric PCBO is illustrated (Figure 3h). The energy bands of PCBO are tilted under US irradiation, generating a built‐in electric field and delaying the recombination of the electron‐hole pairs. The CB position of PCBO was lower than the *E* ^0^ of oxidation of O_2_/·O_2_^−^, confirming that PCBO could make O_2_ obtain e^−^ formation ·O_2_^−^ under US irradiation. Intriguingly, PCBO possesses the ability to deplete GSH using the 5,5′‐dithiobis‐(2‐nitrobenzoic acid) (DTNB) probe due to redox potential of Cu^2+^ ions. The concentration of GSH decreases with increasing co‐incubation time treated with PCBO (Figure 3i; Figure S10, Supporting Information), verifying that PCBO possesses the GSH‐consuming property.

### 2.3 Biocompatibility, Biosafety, and Photoacoustic Imaging of PCBO

**Figure 4:**
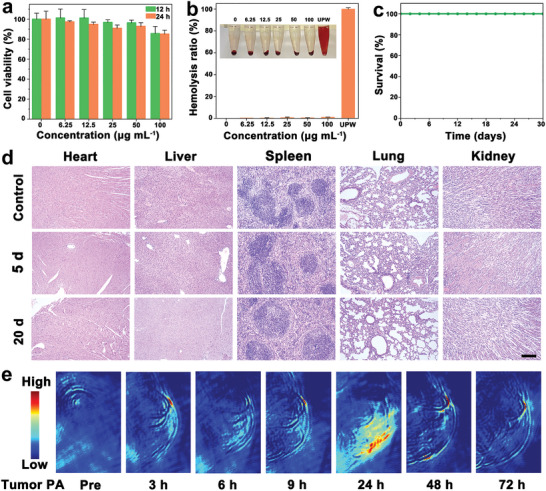
The biosafety and PA imaging of PCBO. a) Cytotoxicity of PCBO at 12 and 24 h in 4T1 cells (*n* = 4). Error bars represent the standard deviation (SD). Data are presented as mean values ± SD. b) Hemolysis of PCBO with different concentrations (ultrapure water (UPW), 0, 6.25, 12.5, 25, 50, and 100 µg mL^−1^). c) Survival curves of healthy mice after intravenous injection with PCBO. d) H&E staining of heart, liver, spleen, lung, and kidney injected with PCBO (15 mg kg^−1^). Scale bar: 200 µm. e) PA images of the tumor‐bearing mice treated with PCBO.

Subsequently, the biocompatibility and biosafety of PCBO were evaluated in detail. First, the standard cell counting kit‐8 (CCK‐8) assay was conducted to evaluate the cytotoxicity (**Figure** **4**a). PCBO exhibits no evident cytotoxicity on 4T1 cells even when incubated with the high concentration (100 µg mL^−1^), which is in agreement with the behavior of other Cu‐based GSH consumes nanomedicine, such as Cu(II)NS, ^[^ ^33^ ^]^ Cu‐HCF nanozyme,^[^ ^34^ ^]^ CuTz‐1‐O_2_@F127,^[^ ^35^ ^]^ and Cu_2_O@Mn_3_Cu_3_O_8_ nanozyme.^[^ ^36^ ^]^ The hemolysis ratio of PCBO was negligible, confirming that PCBO did not trigger the rupture of red blood cells (Figure 4b). Subsequently, the short‐ and long‐term biosafety is evaluated after PCBO intravenous injection into healthy mice. Intriguingly, there is no significant difference in blood routine examination from the PCBO and control groups (Figure S11, Supporting Information). At the same time, all mice were kept alive during the experiment (Figure 4c) the weight change is very similar from the PCBO and control groups (Figure S12, Supporting Information). More importantly, there are also no pathologic cellular changes and inflammatory lesions from the major organs after intravenous injected PCBO by hematoxylin and eosin (H&E) staining (Figure 4d). Notably, PCBO has concentration‐dependent broad absorbance in the near‐infrared (NIR) region (Figure S13, Supporting Information), indicating the potential application in imaging‐mediated therapy. The photoacoustic (PA) images of PCBO dispersions (Figure S14, Supporting Information) and tumor accumulation were monitored (Figure 4e). The PA signal of tumor location can be clearly observed to be significantly enhanced 24 h after intravenous injection. Thus, the appropriate therapeutic window time of the subsequent exploration of anticancer efficacy was set at 24 h after intravenous injection.

### 2.4 Apoptosis Induced by “Three‐Step” with PCBO Transducers

**Figure 5:**
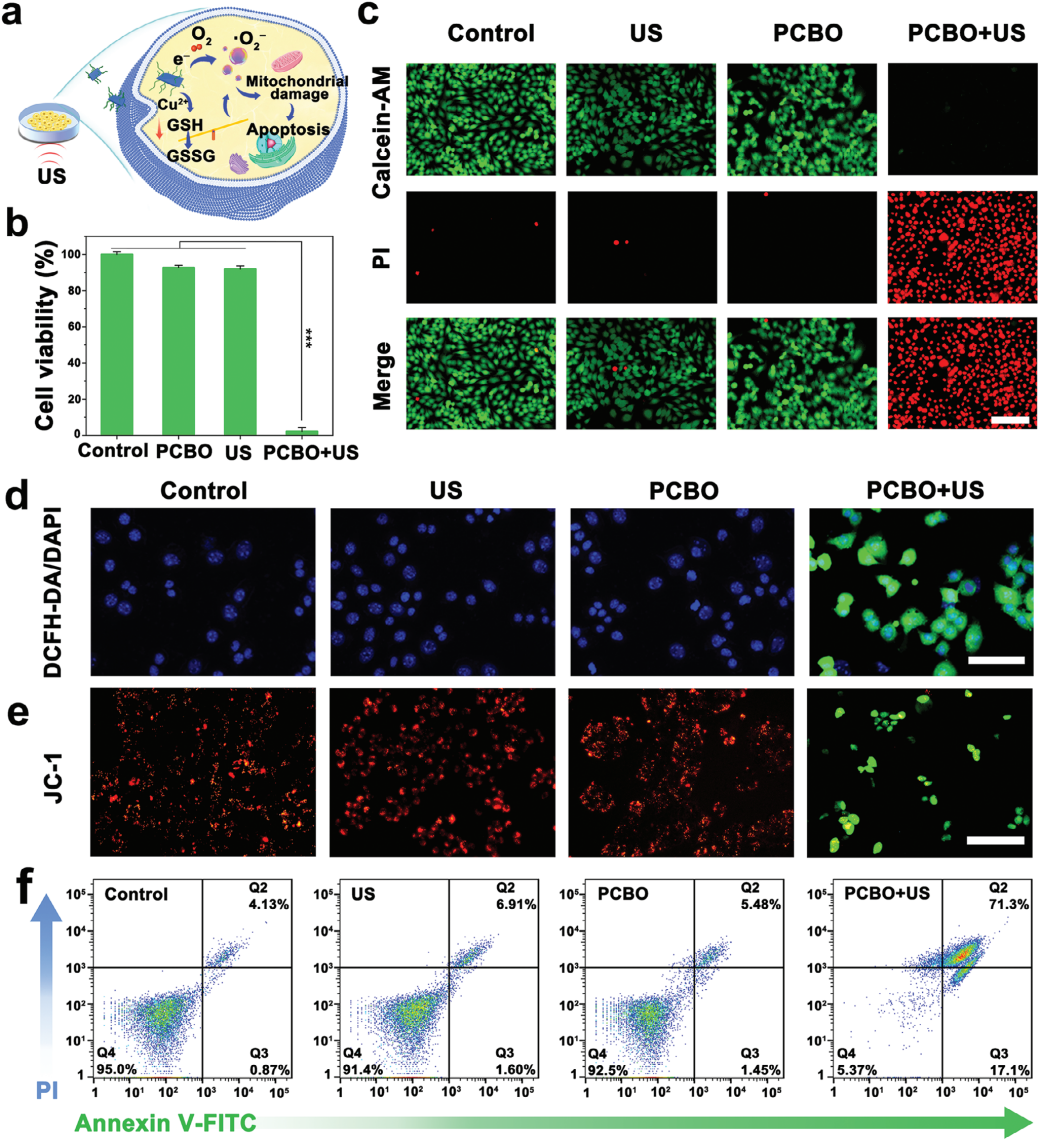
“Three‐step” enhanced SDT efficiency of spatially asymmetric PCBO. a) Illustration showing PCBO promoted energy conversion to realize tumor cell killing. b) Viability of 4T1 cells after various treatments. (*n* = 4) Data are presented as mean values ± SD. Statistical significance was analyzed via a two‐tailed *t*‐test. ^***^ *p* < 0.001. c) Calcein‐AM/PI fluorescence images of 4T1 cells after different treatments. Scale bar: 100 µm. d) Intracellular ROS level treated with different treatments. Scale bar: 50 µm. e). JC‐1 fluorescence images of 4T1 cells after different treatment. Scale bar: 100 µm. f) Assessment of cell apoptosis by Annexin V‐FITC and PI staining.

Intrigued by the attractive piezoelectric performance and favorable biosafety of PCBO, we further demonstrated the efficacy of SDT systematically at the intracellular level (**Figure** **5**a). As shown in Figure 5b, the cell viability was significantly reduced in the PCBO + US group, while cell damage was negligible in the control, PCBO, or US groups, in accordance with the results of live/dead cell stain assay (Figure 5c). For instance, the PCBO + US group exhibits the potent cell‐killing effect (red fluorescence) while the other groups show exuberant cell vitality (green fluorescence). Then, the intracellular ROS was directly observed by the 2′,7′‐dichlorofluorescin diacetate (DCFH‐DA) probe, and 4′,6‐diamidino‐2‐phenylindole, dihydrochloride (DAPI) cell nucleus fluorescent dye. The most intense fluorescence signal of 2′,7′‐dichlorofluorescein (DCF) is discerned in the PCBO + US group while that of the other groups has almost none (Figure 5d; Figure S15, Supporting Information), suggesting that PCBO stimulated by the US could induce the significant generation of ROS. Additionally, 5,5′,6,6′‐tetrachloro‐1,1′,3,3′‐tetraathylimidacarbocyanine iodide (JC‐1) probe was used to detect mitochondrial damage (Figure 5e; Figure S16, Supporting Information). Intriguingly, the emergence of green fluorescence within the PCBO + US group underlines a decline in mitochondrial membrane potential, a distinct signal of programmed cell death. Successfully, as exhibited in Figure 5f, the apoptotic rate in the PCBO + US group was 88.4%, which is in agreement with the other piezoelectric sonosensitizers (Table S2, Supporting Information), suggesting that the built‐in electric field generated by US‐activated PCBO provides a constant supply of electrons to promote ROS, and the abundant ROS reduces the mitochondrial membrane potential, induce mitochondrial dysfunction, and further trigger cell apoptosis.

### 2.5 Tumor Suppression Effects

**Figure 6:**
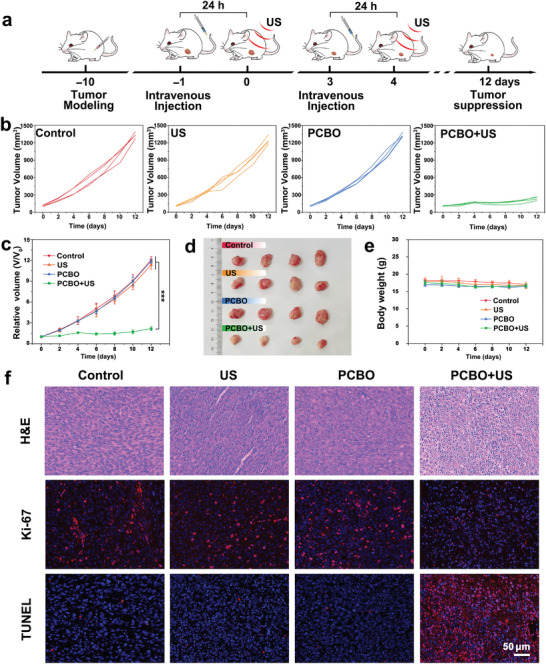
Tumor suppression effects of spatially asymmetric PCBO. a) Treatment illustration of 4T1 tumor‐bearing mice. b) Tumor volume growth curves of 4T1 tumor‐bearing mice after different treatments. c) Relative growth curves of tumors in different groups. Data are presented as mean values ± SD (*n* = 4). Statistical significance was analyzed via a two‐tailed *t*‐test. ^***^ *p* < 0.001. d) Digital tumor photographs from different groups. e) Body weight of 4T1 tumor‐bearing mice after different treatments. Data are presented as mean values ± SD (*n* = 4). f) Staining images of H&E, Ki‐67, and TUNEL on tumor tissues from the aforementioned groups.

Motivated by the decent SDT performance generated from the piezoelectricity of spatially asymmetric PCBO, anticancer therapeutic effects of PCBO were subsequently investigated. Tumor‐bearing mice were randomly divided into PCBO + US, PCBO, US, and control groups, and the SDT process of PCBO was illustrated in **Figure** **6**a. The tumor volume and body weight of mice from the aforementioned groups were recorded every two days during treatment. Interestingly, tumor growth has been significantly inhibited in the PCBO + US group whereas in the other groups, tumors grew inexorably (Figure 6b,c), which could also be confirmed by the tumor photographs at the end of treatment (Figure 6d). Furthermore, the variability of mice body weight is similar in each group (Figure 6e), and no significant tissue damage occurred to major organs such as the heart, liver, spleen, lung, and kidney in the PCBO and PCBO + US groups (Figure S17, Supporting Information), ensuring the safety of treatment.

Successively, the mechanism of tumor suppression was performed by H&E staining, Ki‐67 immunohistochemistry assay, as well as terminal deoxynucleotidyl transferase‐mediated dUTP nick end labeling (TUNEL) staining (Figure 6f). H&E‐staining demonstrates significant changes in the morphology of tumor cells in the PCBO + US group compared to the other three groups. And PCBO + US group exhibited the weak red fluorescence of Ki‐67 antibody staining, demonstrating that the cell proliferation of tumor tissue was substantially inhibited after being treated with PCBO and US stimulation. Meanwhile, TUNEL staining also displayed intense red fluorescence reflecting the DNA damage in the PCBO + US group, prompting cell apoptosis. Therefore, PCBO can effectively and indirectly convert sound energy into chemical energy to kill tumor cells and suppress tumor growth.

## 3 Conclusion

In summary, PCBO with small bandgap and asymmetric structures were designed and manufactured for enhancing SDT. Inspiringly, PCBO have a relatively small bandgap (1.83 eV) due to the hybridization between the Bi 6s and O 2p orbitals in PCBO, facilitating the rapid transfer of charge carriers. Importantly, [CuO_4_]^6−^ layers constructed by nonequivalent Cu atoms and connected by Bi atoms are rotationally stacked along the *c* axis, forming a spatially asymmetric structure that endowed PCBO with fantastic piezoelectric properties. The energy bands of PCBO are tilted under US irradiation, generating a built‐in electric field and delaying the recombination of the electron‐hole pairs. In consequence, abundant electrons driven by the electric field accumulate on the surface of PCBO under US irradiation, ensuring the plentiful generation of ROS. The energy conversion strategy based on PCBO significantly causes the mitochondrial damage, leading to apoptosis and inhibiting tumor proliferation. This work provided PCBO as a piezoelectric sonosensitizers innovatively, broadening piezoelectric sonosensitizers for tumor therapy in the prospect of clinical transformation.

## 4 Experimental Section

### Materials

Cu(NO_3_)_2_·3H_2_O, Bi(NO_3_)_3_·5H_2_O, formamide, GSH, PVP 58 000 and DTNB were purchased from Aladdin. CCK‐8, Calcein AM/PI Cell viability/cytotoxicity assay kit, DCFH‐DA assay kit, and DAPI dihydrochloride were purchased from Beyotime. AnnexinV‐FITC/PI apoptosis detection kit was purchased from Solarbio.

### Synthesis of PCBO

Cu(NO_3_)_2_·3H_2_O (0.217 g) and 0.970 g Bi(NO_3_)_3_·5H_2_O was dispersed in 25 mL nitric acid (0.5 m) and stirred for 1 h. Then, 25 mL of NaOH aqueous solution (2 m) was added to the mentioned solution and stirred for 3 h. The precursor solution was transferred into 50 mL Teflon autoclave and maintained at 180 °C for 12 h to obtain CuBi_2_O_4_ microspheres. Then, 100 mg CuBi_2_O_4_ microspheres were added to 15 mL formamide and pulverized with the ultrasonic homogenizer (5 h, 500 W) to obtain the CBO nanofragments. Subsequently, 100 mg PVP was added into 10 mL of the CBO nanofragments dispersions and ultrasonic (100 W) for 30 min. Finally, PCBO was collected by differential centrifugation.

### Characterization

AC‐HAADF‐STEM image was achieved by Titan Cubed Themis G2300. The phase composition was characterized by Bruker D8 Discover XRD. The Rietveld method of the General Structural Analysis Software System II program was used for structural refinement. Cu K‐edge XANES and EXAFS data were monitored in fluorescence mode on the X‐ray absorption spectroscopy beamline at the Shanghai Synchrotron Radiation Facility.

### Ultrasonic Current Measurement

The ultrasonic current of PCBO was measured through electrochemical work station. Specifically, working electrode (samples), a reference electrode (saturated calomel electro), and an auxiliary (platinum plate) were immersed in the electrolyte solution (Na_2_SO_4_: 0.5 m). The materials were coated on the conductive surface of the working electrode. US irradiation was used as the excitation source of current signal, and a testing cycle was composed of a 20 s ultrasound process and a 20 s silence condition.

### Detection of ·O_2_^−^ Production

A 50 µL of DPBF solutions (1 mg mL^−1^) were added into 3 mL of PCBO solutions (25 µg mL^−1^) and the mixed solutions were exposed to US irradiation (50% duty cycle, 1 W cm^−2^) at different durations. The UV–vis absorption spectra of the mixed dispersions were measured by using a UV‐1900i spectrophotometer.

### ESR Measurement of O_2_^−^

The PCBO dispersions (25 µg mL^−1^) were used to detect ·O_2_^−^ after treatment with US irradiation (1.0 W cm^−2^, 3 min). DMPO (10 µL) was added into PCBO methanol dispersions, and the mixed dispersions were examined by using the ESR.

### Detection of GSH Consumption

PCBO dispersions (10 mL, 25 µg mL^−1^) and GSH (100 µm) were maintained under magnetic agitation (*n* = 5). At different time points (0, 30, 60, 90, and 150 min), 3 mL of the mixed dispersions were added with DTNB indicator, and GSH consumption was monitored by detecting the change of the absorption value at 412 nm.

### Cytotoxicity Assay

4T1 cells (10^4^ cells per well) were seeded in 96‐well cell culture plates and 24 h incubation, fresh cell medium containing PCBO dispersions (0, 6.25, 12.5, 25, 50, 100 µg mL^–1^) was added into wells and coincubated for 24 h. Subsequently, the standard CCK‐8 assay procedure is utilized to evaluate the cytotoxicity of PCBO.

### Live/Dead Assay

4T1 cells (10^4^ per well) were seeded in 96‐well culture plates and incubated for 24 h. Subsequently, the cells were treated with four groups: control, US, PCBO (50 µg mL^−1^), and PCBO + US groups. For instance, the cells were treated with US irradiation after co‐incubated with PCBO for 3 h in PCBO + US groups. Then, the cells from the four groups were incubated for another 21 h. The medium from the four groups was alternated by staining a solution containing calcein‐AM and PI. After incubating for 20 min, the fluorescence images were obtained from an inverted fluorescence microscope.

### Detection of Intracellular ROS Production

4T1 cells (10^4^ per well) were seeded in 96‐well culture plates and incubated for 24 h. Subsequently, the cells were treated with four groups: control, US, PCBO (50 µg mL^−1^) and PCBO + US groups (50 µg mL^−1^). For instance, the cells in PCBO + US groups were co‐incubated with PCBO for 3 h, DCFH‐DA, and DAPI probes were added into the medium and the obtained medium was treated with the US irradiation. After incubating for 20 min, the fluorescence images were obtained from inverted fluorescence microscope.

### Mitochondrial Membrane Potential Detection

4T1 cells (10^4^ per well) were seeded in 96‐well culture plates and incubated for 24 h. Subsequently, the cells were treated with four groups: control, US, PCBO (50 µg mL^−1^), and PCBO + US groups. For instance, the cells were treated with US irradiation after co‐incubated with PCBO for 3 h in PCBO + US groups. The cells from the four groups were incubated for other 4 h. Finally, JC‐1 was used as the indicated probe for staining through the manufacturer's standard procedure.

### Apoptosis Detection Assay

The 4T1 cells (10^5^ cells per well) were seeded in 6‐well plates and incubated for 24 h. Subsequently, the cells were treated with four groups: control, US, PCBO, and PCBO + US groups. For instance, the cells in PCBO + US groups were treated with PCBO (25 µg mL^−1^), and US treatment was operated after co‐incubated for 3 h. After treatment, cells from four groups were incubated for 21 h and then were detached by trypsin and added an Annexin V‐FITC/PI apoptosis detection kit to analyze quantitatively apoptosis‐mediated cell death by flow cytometry.

### Tumor Model

The protocols for all in vivo experiments were approved by the Animal Care Ethics Commission of Shanghai Rat&Mouse Biotech Company Limited (approval No. SHRM‐ IACUC‐059). The 4T1 cells were subcutaneously injected into the right hind legs of a female Balb/c mouse for the tumor model.

### Biocompatibility

The healthy Balb/c mice were intravenously injected with PCBO (15 mg mL^−1^). Blood samples of mice were collected for routine blood tests at 3, 7, and 30 days after injection, respectively. During this period, body weight of mice was recorded every 2 days. In addition, H&E staining was performed on major organs including heart, liver, spleen, lung, and kidney on 5 and 20 days.

### PA Imaging

Tumor bearing mice were intravenously injected with PCBO (100 µL, 15 mg mL^−1^), the mice were scanned by PA imaging system before injection and at different times (0, 3, 6, 9, 24, 48, and 72 h) after injection.

### SDT Effects In Vivo

Tumor bearing mice were randomly divided into PCBO + US, PCBO, US, and control groups. PCBO + US group was intravenously injected twice (100 µL, 15 mg kg^−1^) and received corresponding US irradiation treatment (2.5 W cm^−2^, 10 min). During treatment, the tumor volume and body weight of mice were recorded every two days. Tumor volume was calculated by the formula: *V*_tumor _= (W^2 ^× L)/2, in where W represents the width of the tumor and L represents the length of the tumor. After 12 days, the tumors obtained from the four groups were collected and stained with H&E, TUNEL, and Ki‐67 antibody staining.

### Statistical Analysis

The quantitative data analysis was presented as mean ± standard deviation (SD). Statistical analysis was performed via a two‐tailed *t*‐test. The *p* values < 0.05 were considered statistically significant (^∗^ *p* < 0.05, ^∗∗^ *p* < 0.01, ^∗∗∗^ *p* < 0.001).

## Conflict of Interest

The authors declare no conflict of interest.

## Supporting information

Supporting Information

## Data Availability

Research data are not shared.
