# Supplementary material for: Novel Spatially Asymmetric Copper Bismuthate‐Mediated Augmentation of Energy Conversion to Realize “Three‐Step” Tumor Suppression
Source: Adv Sci (Weinh). 2024 Apr 23;11(25):2402599. doi: 10.1002/advs.202402599 (PMC11220698; doi:10.1002/advs.202402599)
Supplement: Supplementary file 1 — Supporting Information [file ADVS-11-2402599-s001.pdf]

ADVANCED SCIENCE
Open Access

Supporting Information

for *Adv. Sci.*, DOI 10.1002/advs.202402599

Novel Spatially Asymmetric Copper Bismuthate-Mediated Augmentation of Energy Conversion to Realize "Three-Step" Tumor Suppression

Jiarui Wang, Haoqin Zheng, Guangyao Hu, Xujian Yang, Hongpeng You, Lile Dong and Shuyan Song**

Copyright WILEY-VCH Verlag GmbH & Co. KGaA, 69469 Weinheim, Germany, 2020.

Supporting Information

Novel Spatially Asymmetric Copper Bismuthate-Mediated Augmentation of Energy Conversion to Realize "Three-Step" Tumor Suppression

Jiarui Wang, Haoqin Zheng, Guangyao Hu, Xujian Yang, Hongpeng You, Lile Dong and Shuyan Song,**

J. Wang, H. Zheng, G. Hu, X. Yang, Prof. H. You, and L. Dong
Key Laboratory of Rare Earths, Chinese Academy of Sciences, Ganjiang Innovation Academy, Chinese Academy of Sciences, Ganzhou 341000, China
School of Rare Earths, University of Science and Technology of China, Hefei 230026, China
E-mail: lldong@gia.cas.cn

Prof. S. Song
State Key Laboratory of Rare Earth Resource Utilization, Changchun Institute of Applied Chemistry, Chinese Academy of Sciences, Changchun 130022, China
Email: songsy@ciac.ac.cn

Keywords: $CuBi_2O_4$, spatially asymmetric, sonosensitizers, tumor suppression, sonodynamic therapy

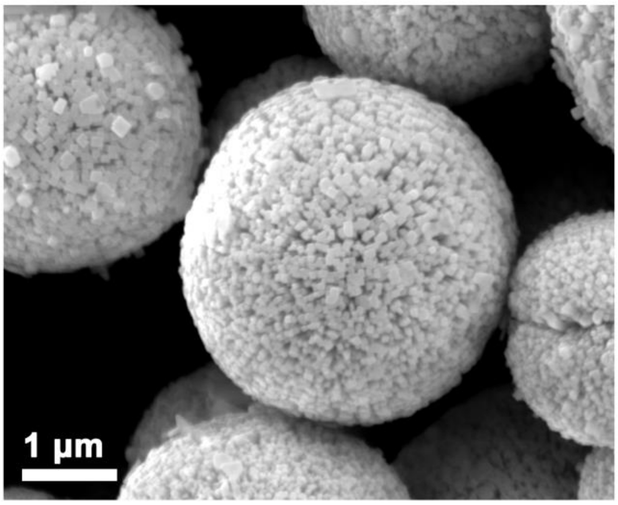

Figure S1. Scanning electron microscope (SEM) images of $CuBi_2O_4$ microspheres.

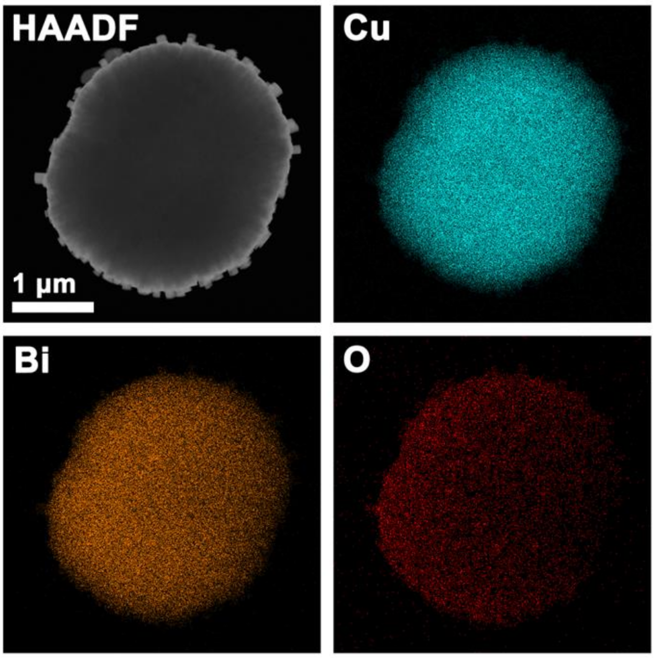

Figure S2. Energy-dispersive X-ray spectroscopy (EDX) element mapping images of $CuBi_2O_4$ microspheres.

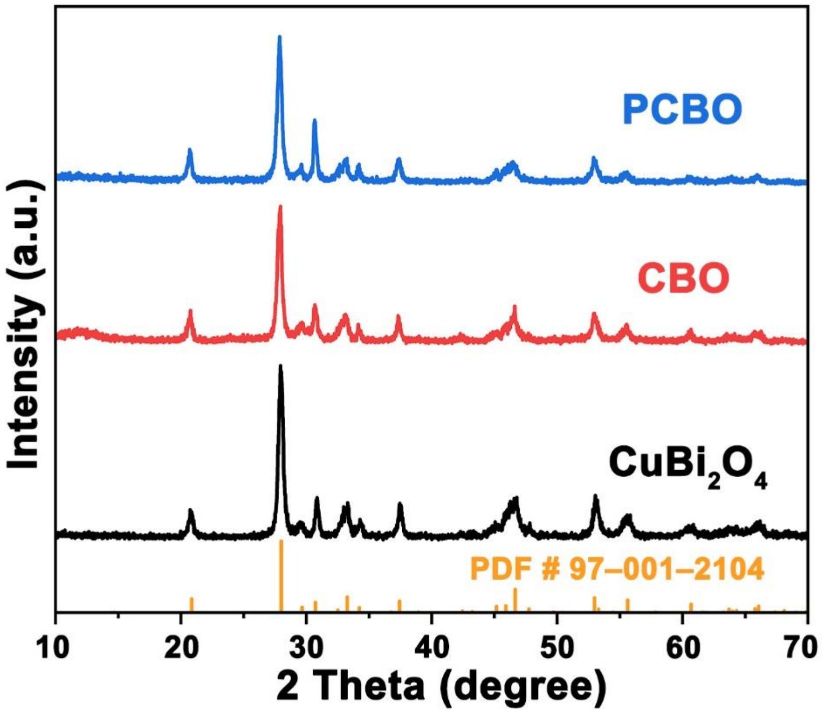

Figure S3. X-ray diffraction (XRD) patterns of PCBO, CBO and $CuBi_2O_4$ microspheres.

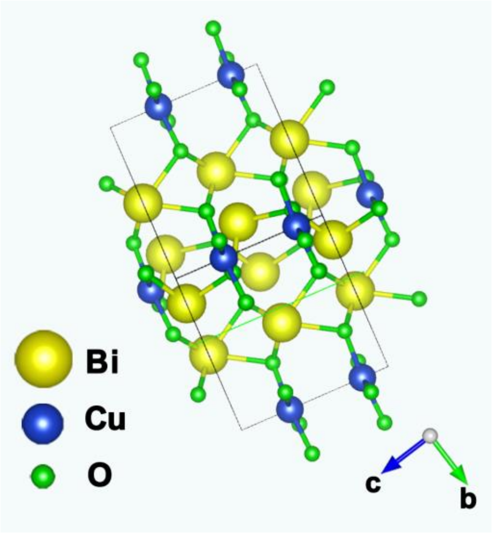

Figure S4. The spatially asymmetric structure sketch of PCBO.

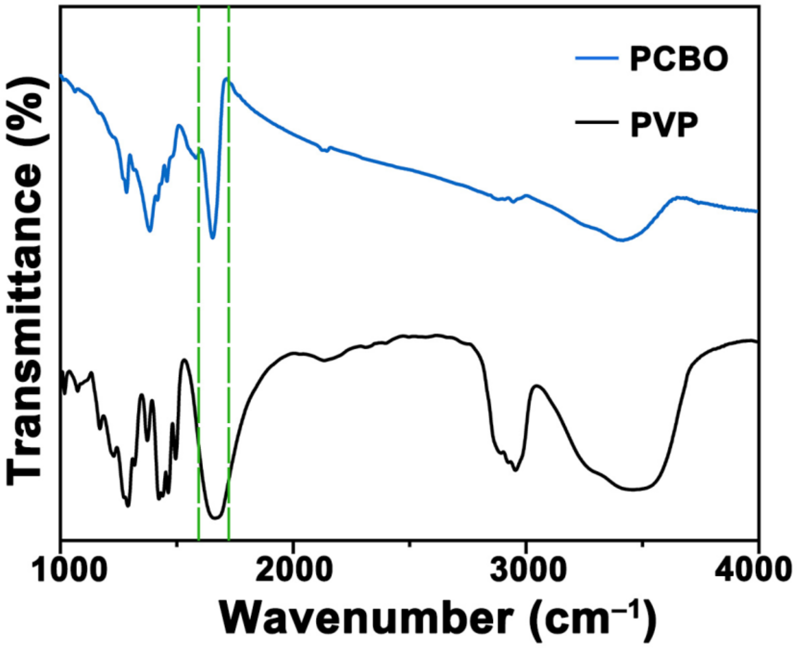

Figure S5. Fourier transform infrared (FTIR) spectroscopy of PCBO piezoelectric sonosensitizers and PVP.

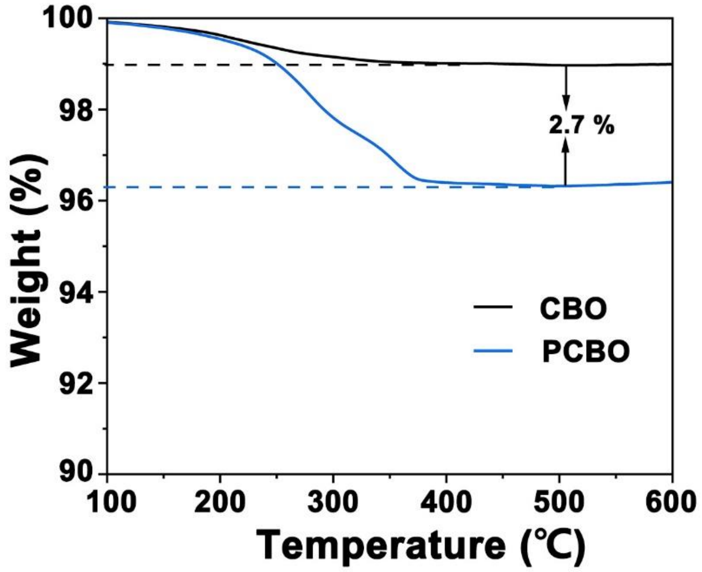

Figure S6. Thermogravimetric analysis (TGA) curves of CBO microspheres and PCBO.

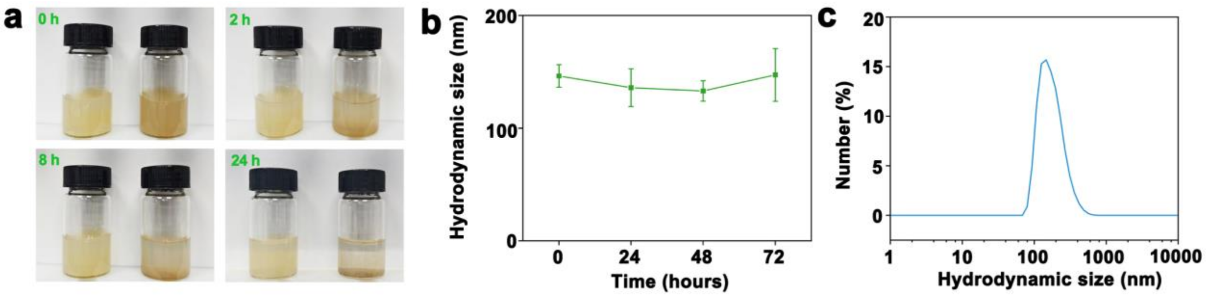

Figure S7. a) The photographs of PCBO (left) or CBO (right) dispersions. b) The changes of hydrodynamic size of PCBO dispersions. c) Hydrodynamic size of PCBO dispersions after 30 days.

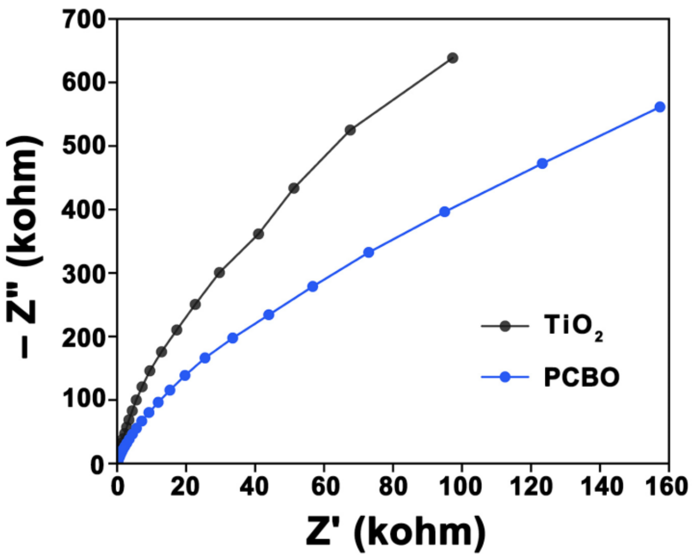

Figure S8. Electrochemical impedance spectra of TiO_2 and PCBO.

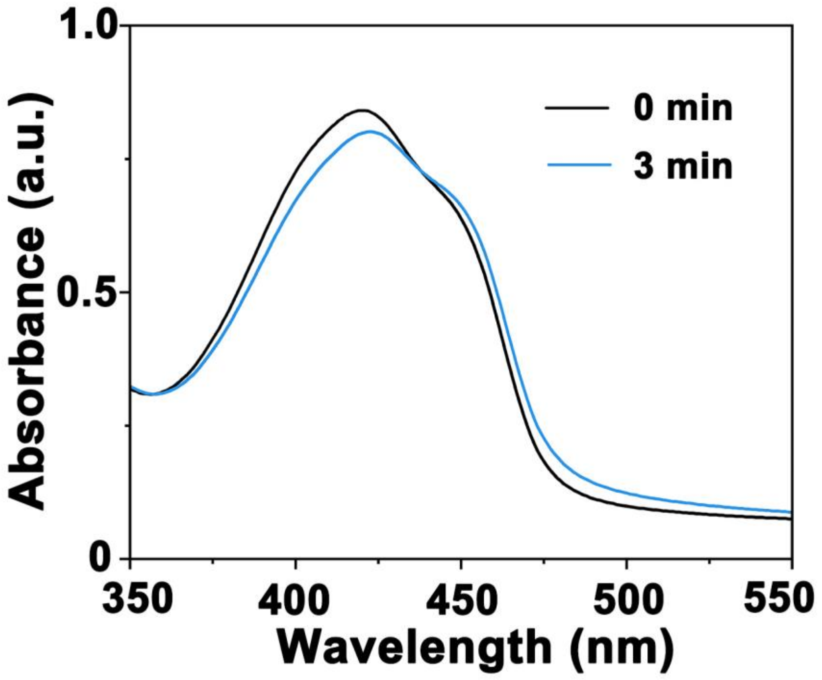

Figure S9. UV-vis absorption spectra of DPBF with or without US irradiation.

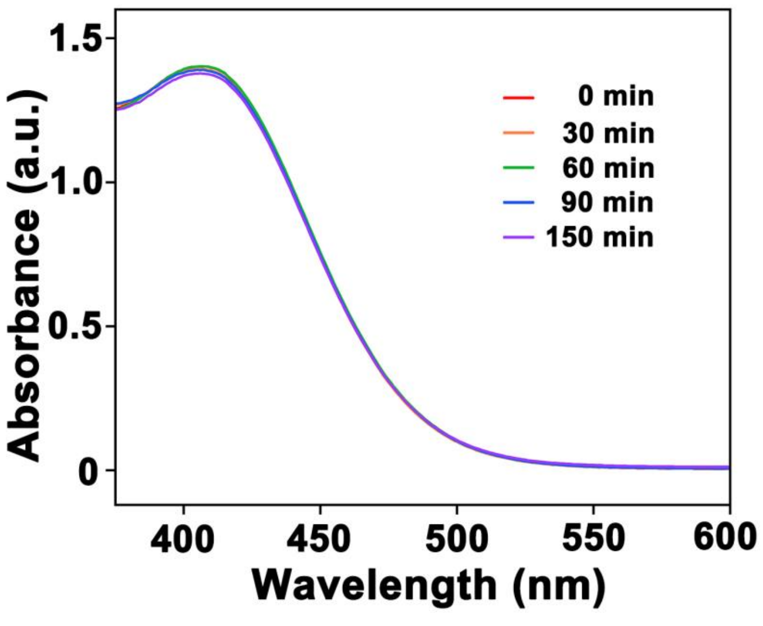

Figure S10. DTNB absorbance versus time in the control group.

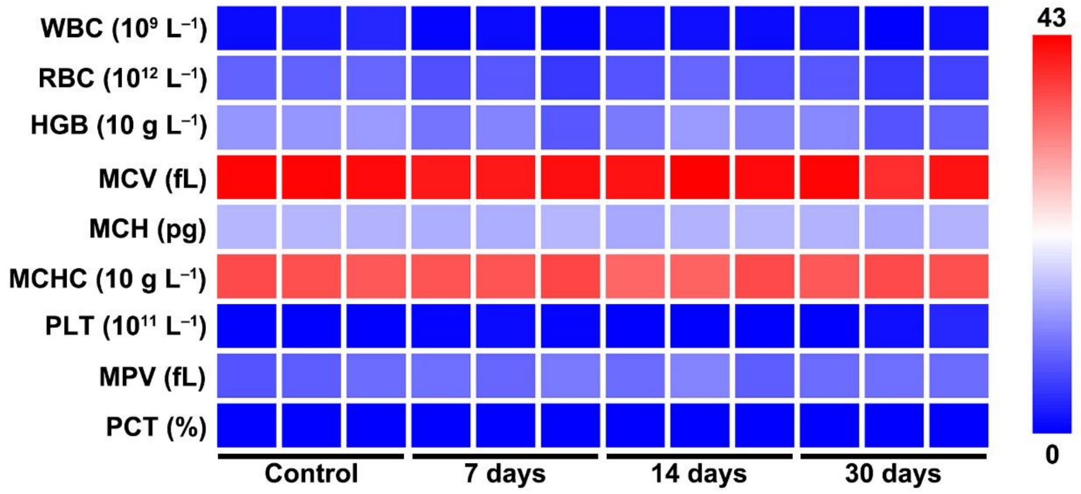

Figure S11. Blood routine indexes of healthy mice from the PCBO-treated groups and control group. n=3 independent experiments per group.

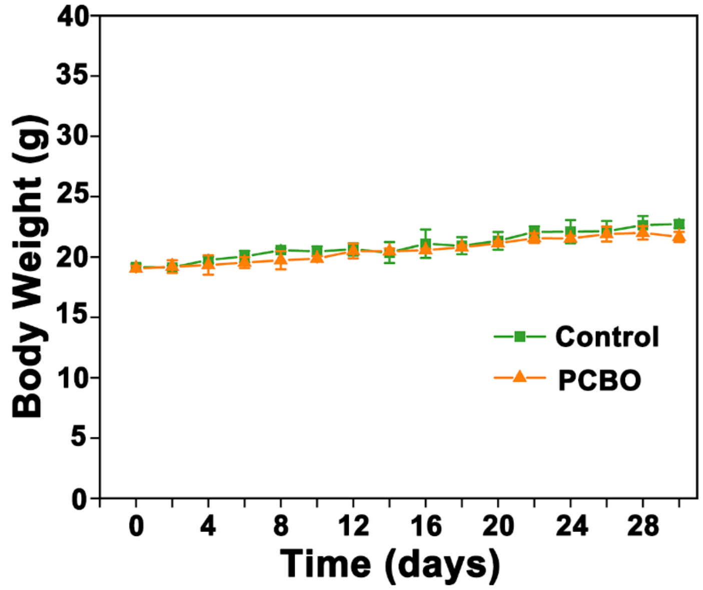

Figure S12. Body weight of Balb/c mice after injection of PCBO.

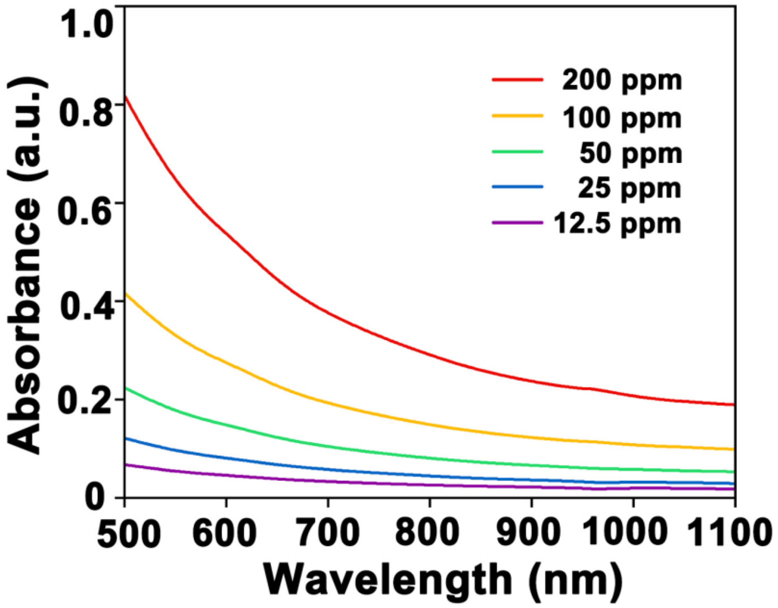

Figure S13. NIR-vis absorbance spectra of different concentrations of PCBO.

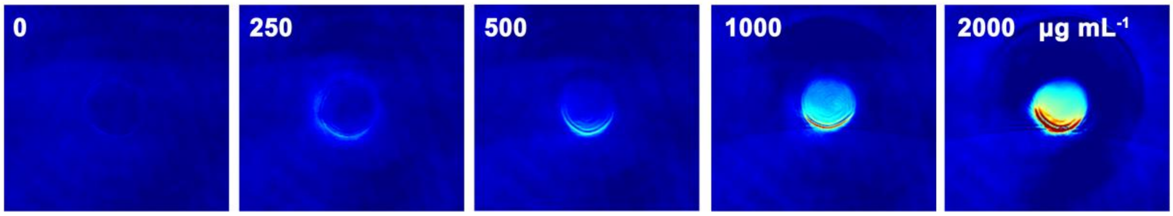

Figure S14. PA images of a series of PCBO dispersions.

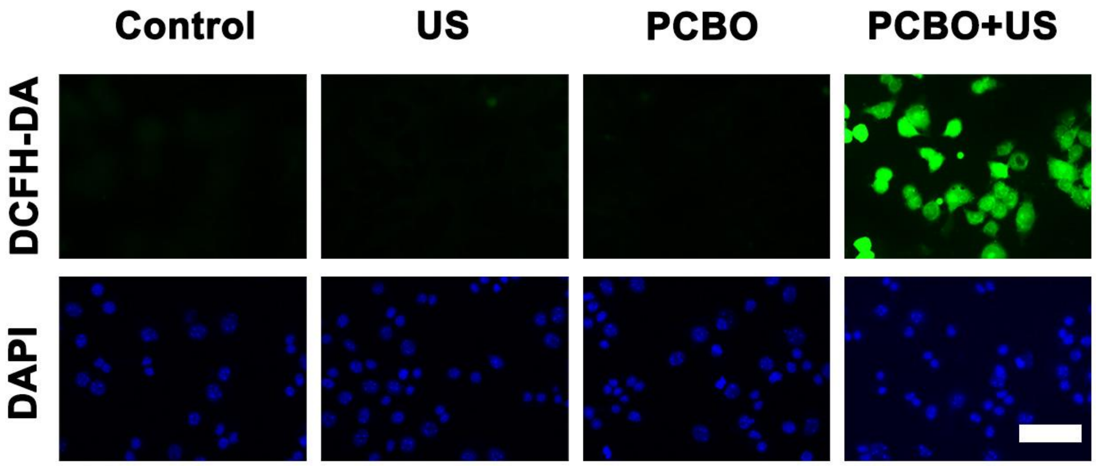

Figure S15. Intracellular ROS level treated with different treatments. Scale bar: 50 μm.

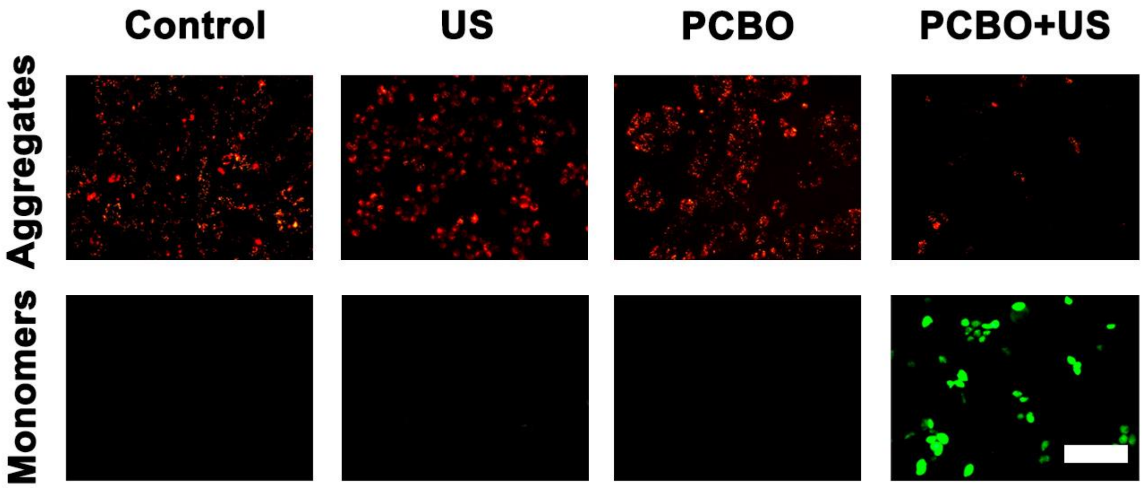

Figure S16. JC-1 fluorescence images of 4T1 cells after different treatment. Scale bar: 100 μm

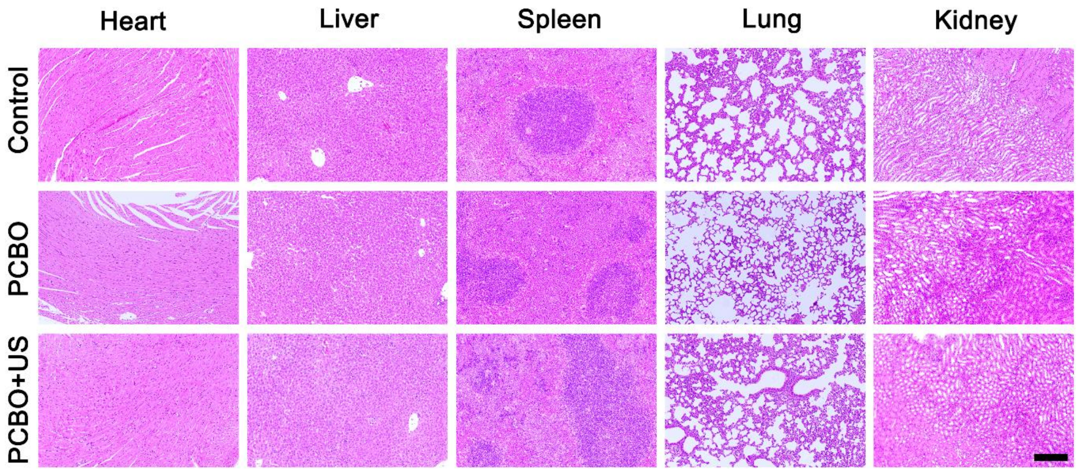

Figure S17. H&E staining of heart, liver, spleen, lung, and kidney after treatments of control, PCBO and PCBO+US. Scale bar: 200 μm.

Table S1. Rietveld refinement and crystallographic data for PCBO piezoelectric sonosensitizers.

Formula	PCBO
Space group	I4
Crystal system	Tetragonal
a = b (Å)	8.51
c (Å)	5.80
α = γ = β (°)	90
V (Å^3)	419.81
Z	4
R_{wp} (%)	8.11
R_p (%)	6.3
x^2	3.97

Table S2. Representative piezoelectric sonosensitizers.

Paradigm	Bandgap	Apoptosis Rate	References
FX11@TPEG-WS_2	2.30 eV	68.42%	1
Mg-HAP@MS/ONC201	5.3 eV	54.73%	2
Mn-ZnO	2.95 eV	67.2%	3
ATO@Bi-HJ	2.75 eV	81.73%	4
G-Bi_2MoO_6	2.97 eV	72.0%	5
Au-ZnO	2.95 eV	78.24%	6
PCBO	1.83 eV	88.4%	This work

References

Q. Hoang, K. Huynh, T. Cao, J. Kang, X. Dang, V. Ravichandran, H. Kang, M. Lee, J. Kim, Y. Ko, T. Lee, M. Shim, *Adv. Mater.* **2023**, *35*, 2300437.
J. Yang, Y. Du, Y. Yao, Y. Liao, B. Wang, X. Yu, K. Yuan, Y. Zhang, F. He, P. Yang, *Adv. Sci.* **2024**, *11*, 2307130.
B. Tian, R. Tian, S. Liu, Y. Wang, S. Gai, Y. Xie, D. Yang, F. He, P. Yang, J. Lin, *Adv. Mater.* **2023**, *35,* 2304262.
X. Cao, Y. Wang, X. Song, W. Lou, X. Li, W. Lu, K. Chen, L. Chen, Y. Chen, B. Huang, *Adv. Funct. Mater.* **2023**, *33,* 2300777.
Y. Dong, S. Dong, B. Liu, C. Yu, J. Liu, D. Yang, P. Yang, J. Lin, *Adv. Mater.* **2021**, *33*, 2106838.
J. Cheng, W. Pan, Y. Zheng, J. Zhang, L. Chen, H. Huang, Y. Chen, R. Wu, *Adv. Mater.* **2024**, 2312102. DOI.org/10.1002/adma.202312102.
